# Development of pyramided lines carrying brown planthopper resistance genes in the genetic background of Indica Group rice (*Oryza sativa* L.) variety ‘IR64’

**DOI:** 10.1270/jsbbs.23028

**Published:** 2023-10-31

**Authors:** Md. Mostofa Kamal, Cuong Dinh Nguyen, Sachiyo Sanada-Morimura, Shao-Hui Zheng, Daisuke Fujita

**Affiliations:** 1 The United Graduate School of Agricultural Sciences, Kagoshima University, 1-21-24 Korimoto, Kagoshima 890-0065, Japan; 2 Agrotechnology Discipline, Khulna University, Khulna 9208, Bangladesh; 3 Biotechnology Department, College of Food Industry, 101B Le Huu Trac Street, Son Tra District, Da Nang City 550000, Vietnam; 4 Agro-Environment Research Division, Kyushu Okinawa Agricultural Research Center, NARO, 2421 Suya, Koshi, Kumamoto 861-1192, Japan; 5 Faculty of Agriculture, Saga University, 1 Honjo-machi, Saga 840-8502, Japan

**Keywords:** marker-assisted selection, gene pyramiding, virulent BPH, ‘IR64’

## Abstract

The development of resistant rice (*Oryza sativa* L.) varieties is a key strategy for the eco-friendly control of brown planthopper (BPH: *Nilaparvata lugens* Stål). However, BPH outbreaks occur frequently owing to the evolution of virulent strains in the field and the rapid breakdown of monogenic resistance to BPH. Therefore, to enhance BPH resistance and gauge the effectiveness of gene pyramiding against strongly virulent BPH, we developed pyramided lines (PYLs) in the genetic background of ‘IR64’ carrying BPH resistance genes. We developed six IR64-PYLs (*BPH3* + *BPH17*, *BPH32* + *BPH17*, *BPH32* + *BPH20*, *BPH3* + *BPH17-ptb*, *BPH20* + *BPH3*, and *BPH17-ptb* + *BPH32*) through marker-assisted selection. To assess the resistance of the IR64-PYLs, we conducted antibiosis test, honeydew test, and modified seedbox screening test (MSST) using strongly virulent BPH populations. The level of BPH resistance increased in all six IR64-PYLs compared to both ‘IR64’ and the corresponding NILs in MSST. Among them, IR64-*BPH3* + *BPH17* and IR64-*BPH32* + *BPH17* exhibited the highest resistance to BPH. However, the resistance level of most IR64-PYLs was not significantly higher than that of the corresponding NILs in antibiosis test. Thus, these PYLs could serve as a valuable resource for breeding programs aimed at improving resistance to virulent strains of BPH and enhancing their durability.

## Introduction

The brown planthopper (BPH: *Nilaparvata lugens* Stål) is a major threat to sustainable rice (*Oryza sativa* L.) production in Asia. BPH not only produces ‘hopper burn’ symptoms as direct damage due to heavy infestation in paddy fields, but also hampers rice production indirectly through the transmission of viral diseases such as *Rice ragged stunt virus* and *Rice grassy stunt virus* diseases (Wei *et al.* 2018). BPH outbreaks caused severe damage in Japan and Korea in 2005, in China in 2005–2007, in Central Thailand in 2009, and in Indonesia in 2011 and 2014 (Jena *et al.* 2017, Li *et al.* 2019). Rice farmers rely on chemical control of BPH, but such measures are not eco-friendly. The use of host-plant resistance offers a lower-cost, environmentally friendly way to mitigate BPH problems in field conditions.

Bred conventionally, the resistant variety ‘IR26’, carrying resistance gene *BPH1*, was released in 1973 and widely grown in the Philippines, Indonesia, and Vietnam, but within a few years, BPH biotype 2 overcame its resistance (Cohen *et al.* 1997). In 1976, to enhance resistance to biotype 2, ‘IR36’, containing resistance gene *BPH2*, was released, but BPH overcame its resistance in 1982 (Alam and Cohen 1998, Khush and Virk 2005). Lines and varieties carrying a single resistance gene became susceptible to current strong BPH populations (Fujii *et al.* 2021, Myint *et al.* 2012, Nguyen *et al.* 2019). Therefore, lines and varieties with a single resistance gene are not durable, because it breaks down quickly with the emergence of new virulent BPH.

In response to severe damage and frequent outbreaks of BPH in several rice-growing regions, pyramiding of multiple resistance genes into elite rice variety has been used to develop durable resistance. Pyramiding of resistance genes or QTLs resulted in stable and long-lasting resistance against BPH (Alam and Cohen 1998). It is also effective against the rapid adaptation by BPH to resistant varieties (Horgan 2018). Pyramided lines (PYLs) offered greater resistance than that of lines harboring single genes against BPH (Qiu *et al.* 2012). PYLs with *BPH6* and *BPH12* had higher resistance than lines containing either one alone. The introduction of *BPH14* and *BPH15* into ‘Minghui 63’, ‘Huahui 938’, and ‘Huang-Hua-Zhan’ increased resistance (Han *et al.* 2018). PYLs carrying *BPH3* and *BPH27(t)* in the ‘Ningjing 3’ and ‘9311’ background (Liu *et al.* 2016) and a PYL carrying *BPH3*, *BPH14*, and *BPH15* in the ‘Hemeizhan’ background had higher resistance than that of NILs containing single genes (Hu *et al.* 2016). A PYL carrying *BPH14*, *BPH15*, and *BPH18* in Indica rice ‘93-11’ had the highest resistance (Hu *et al.* 2013).

The elite Indica Group variety ‘IR64’ has been widely grown in South and Southeast Asia since the 1980s (Khush and Virk 2005) for its good eating quality and high yields. The variety ‘IR64’ carrying *BPH1* and *BPH37* had moderate resistance to BPH but became susceptible in Indonesia during 1990s (Yang *et al.* 2019). BPH populations that migrated recently into Japan have already overcome resistance conferred by the single genes *BPH1*, *BPH2*, *BPH3*, *BPH17*, *BPH20*, and *BPH32* (Fujii *et al.* 2021, Nguyen *et al.* 2019). However, these genes might still be able to enhance BPH resistance through gene pyramiding. Therefore, the objective of this study is to develop PYLs and evaluate the pyramiding effect of four resistance genes in the ‘IR64’ genetic background. We characterized these PYLs against two BPH populations (Koshi-2013 and Koshi-2020, which recently migrated from China into Japan) to confirm their effectiveness, even though each single resistance gene has lost its effect.

## Materials and Methods

### Plant materials for the development of PYLs with BPH resistance genes

**Fig. 1.**
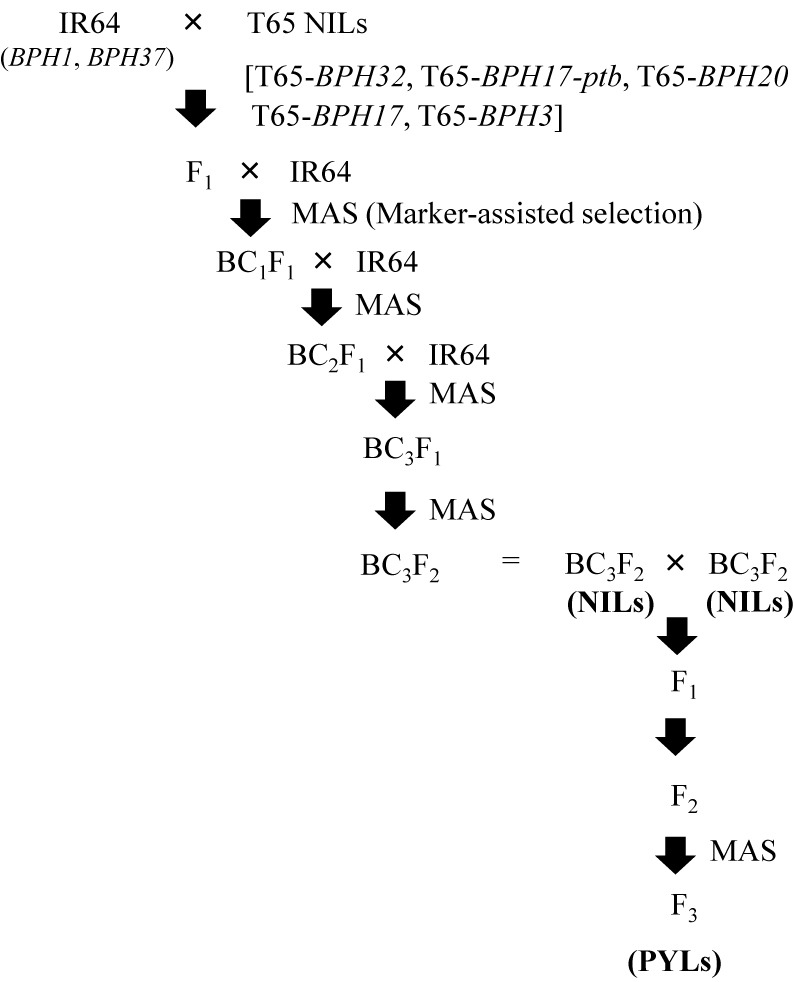
Breeding scheme for the development of pyramided lines (PYLs) with BPH resistance genes in the genetic background of Indica Group variety ‘IR64’.

Five IR64-NILs—IR64-*BPH32*, IR64-*BPH17-ptb*, IR64-*BPH20*, IR64-*BPH17*, and IR64-*BPH3* (Kamal *et al.* 2023) were used for the development of the PYLs. PYLs for two resistance genes were developed from IR64-NILs descended from the BC_3_F_2_ generation (Fig. 1). The F_1_ plants derived from crosses between NILs were self-pollinated to produce F_2_ plants. From 96 F_2_ plants, plants that were homozygous for the two BPH resistance genes were selected by marker-assisted selection (MAS) using SSR markers (Supplemental Table 1). We developed six PYLs—IR64-*BPH3* + *BPH17*, IR64-*BPH32* + *BPH17*, IR64-*BPH32* + *BPH20*, IR64-*BPH3* + *BPH17-ptb*, IR64-*BPH20* + *BPH3*, and IR64-*BPH17-ptb* + *BPH32*—carrying four BPH resistance genes.

### DNA extraction and MAS for BPH resistance genes

We extracted total DNA by the potassium acetate method (Dellaporta *et al.* 1983). The genotype of each plant was determined by PCR and agarose gel electrophoresis as described by Kamal *et al.* (2023). In MAS for resistance genes on chromosome 4S, the plants with *BPH17*, *BPH17-ptb*, and *BPH20* were selected with SSR markers RM8213, MS10, and RM16535. In MAS for genes on chromosome 6S, *BPH32* was selected with SSR markers RM508 and RM19341 and *BPH3* with RM508 and RM588 (Supplemental Table 1).

### Verification of the presence of *BPH17*

We used InDel markers to confirm *BPH17* in the NILs and PYLs (He *et al.* 2020). *BPH17* was cloned as a gene cluster within a segment of <50 kb (6.93–6.98 Mbp) on the short arm of chromosome 4 (Liu *et al.* 2015). We conducted a polymorphism test between ‘IR64’ and the donor parent using eight InDel markers (Supplemental Table 2). Markers I531 and I729 showed polymorphism between the parents. Using these two markers, we confirmed the presence of *BPH17*.

### Brown planthopper populations

BPH populations, Koshi-2013 and Koshi-2020 were collected from Koshi city, Kumamoto Prefecture, Japan, in 2013 and 2020, respectively. Kyushu Okinawa Agricultural Research Center, National Agriculture and Food Research Organization (NARO), Kumamoto, Japan provided both populations, which were subsequently maintained on the susceptible rice variety ‘T65’ (Taichung 65) at 25°C with 16 h/8 h of light/dark at Saga University for the characterization of the resistance of the PYLs.

### Characterization of PYLs against BPH populations

#### Antibiosis test

We used the antibiosis test to evaluate adult mortality as described by Myint *et al.* (2009). Five plants of each PYL, the corresponding NILs, and the parental lines (‘IR64’, ‘Rathu Heenati’, and ‘T65’) were separately grown in 215-mL plastic cups. All seedlings were trimmed to 15 cm height at 4 weeks and placed in a plastic case. Each plant was infested with 5 thin-abdomen brachypterous female BPH. At 5 days after infestation (DAI), adult mortality (i.e., the number of dead adults on each plant) was calculated.

#### Honeydew test

Areas of honeydew were measured by the method of Heinrichs *et al.* (1985) with modifications. Seedlings ~30 days old were grown in 215-mL plastic cups. Filter paper treated with 0.2% bromocresol green was placed inside a ventilated plastic chamber to absorb the plant honeydew excreted by BPH. After BPH were starved for 1 h, 2 large-abdomen brachypterous females were placed inside the chamber on each plant. Filter papers were collected after 24 h, and honeydew areas were measured in ImageJ v. 1.53a software.

#### Modified seedbox screening test

To evaluate the resistance of PYLs, we performed the modified seedbox screening test (MSST) (Horgan *et al.* 2015). Twenty-five seeds of each PYL, the corresponding NILs, ‘Rathu Heenati’, ‘IR64’ and ‘T65’ were sown per row in a plastic tray (23.0 × 30.0 × 2.5 cm) with 2.5 cm between rows. Seven days after sowing (DAS), the plants were thinned to 20 per row and infested with second and third instar nymphs at a density of around 20 nymphs per plant. When all ‘T65’ plants had dried, the damage scores (DSs) of all lines and varieties were classified according to the standard evaluation system established by IRRI (IRRI 2014).

### Characterization of PYLs for agronomic traits

The IR64-PYLs and ‘IR64’ were grown in a paddy field at Saga University in 2022. Seedlings were transplanted at 30 DAS, at one plant per hill, with 20 cm between hills and 25 cm between rows. We recorded days to heading (DTH), culm length (CL), panicle length (PL), flag leaf length (LL), flag leaf width (LW), and panicle number (PN) of six plants in a row as described by Nguyen *et al.* (2019).

## Results

### Development of PYLs with BPH resistance genes in the ‘IR64’ background

From F_3_ generations, we selected six PYLs for evaluating BPH resistance: IR64-*BPH3* + *BPH17*, IR64-*BPH32* + *BPH17*, IR64-*BPH32* + *BPH20*, IR64-*BPH3* + *BPH17-ptb*, IR64-*BPH20* + *BPH3*, and IR64-*BPH17-ptb* + *BPH32* (Table 1).

### Adult mortality of BPH on IR64-PYLs

Using Koshi-2013, we assessed the resistance of the six IR64-PYLs by antibiosis test using adult mortality at 5 DAI (Table 2). The adult mortality on ‘IR64’ was 48.0% and that on the six IR64-PYLs ranged from 92.0% to 100.0%. However, that on IR64-*BPH32* + *BPH17*, IR64-*BPH3* + *BPH17-ptb* and IR64-*BPH17-ptb* + *BPH32* were significantly higher than that of at least one corresponding IR64-NIL against Koshi-2013.

We also evaluated the adult mortality of Koshi-2020 (Table 2). The adult mortality on ‘IR64’ was 45.0% and that on the six IR64-PYLs ranged from 80.0% to 95.0%. That on IR64-PYLs was non-significantly higher than that on the corresponding IR64-NILs against Koshi-2020.

### Honeydew excretion of BPH on IR64-PYLs

We assessed the reaction of the Koshi-2013 feeding rate to the six IR64-PYLs on the basis of honeydew excretion area (Table 3, Supplemental Fig. 1B). The area of honeydew excreted ranged from 2.6 to 6.2 mm^2^ and was significantly lower than that on ‘IR64’ (26.3 mm^2^). The area of honeydew on IR64-PYLs was slightly lower than that on the corresponding IR64-NILs, but not significantly different from most of IR64-PYLs. However, that on IR64-*BPH3* + *BPH17-ptb* and IR64-*BPH17-ptb* + *BPH32* were significantly lower than that of at least one corresponding IR64-NIL. We also assessed the feeding rate of Koshi-2020 (Table 3). The area of honeydew on the IR64-PYLs ranged from 6.0 to 9.6 mm^2^ and was significantly lower than that on ‘IR64’ (28.4 mm^2^). It was non-significantly lower than on the corresponding IR64-NILs.

### Damage scores of PYLs in MSST

**Fig. 2.**
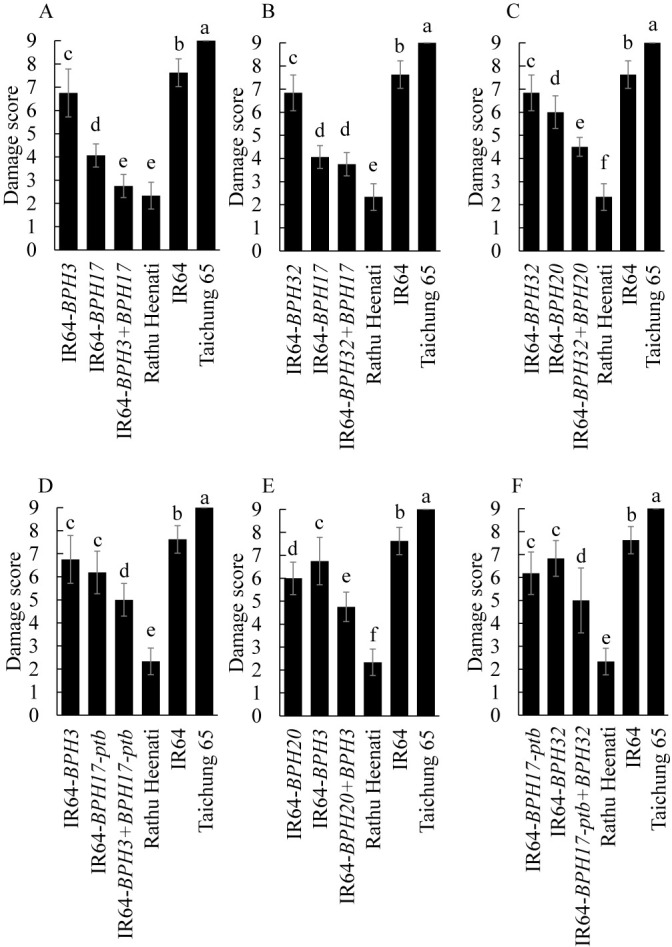
Damage scores of IR64-PYLs by Koshi-2013 in modified seedbox screening test. Values with the same letter are not significantly different at *P* < 0.05 by Tukey–Kramer test.

**Fig. 3.**
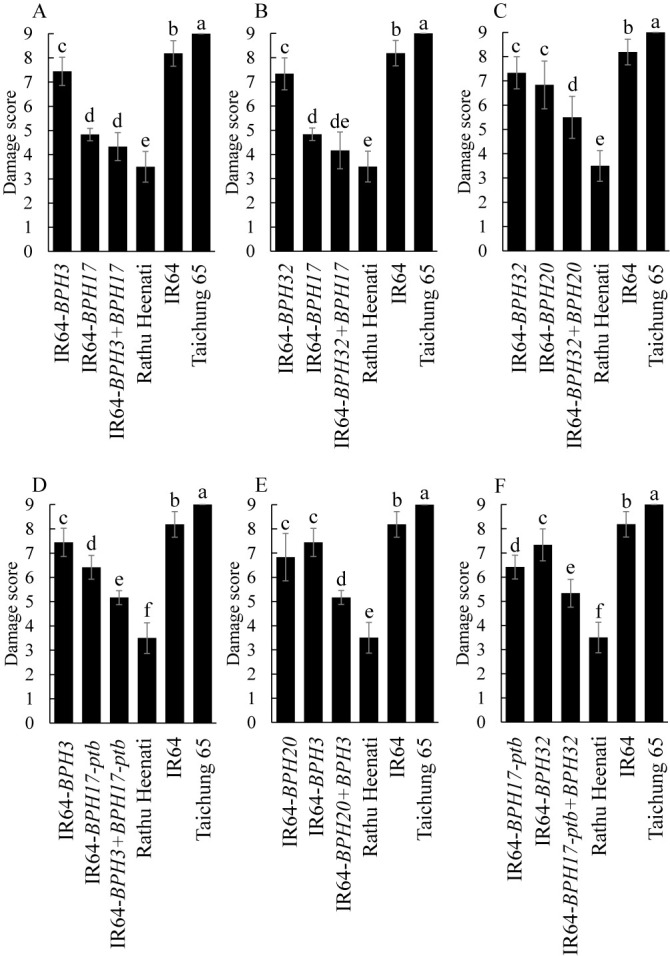
Damage scores of IR64-PYLs by Koshi-2020 in modified seedbox screening test. Values with the same letter are not significantly different at *P* < 0.05 by Tukey–Kramer test.

To characterize resistance of IR64-PYLs, we evaluated the DSs of IR64-PYLs against Koshi-2013 by the MSST (Fig. 2, Supplemental Fig. 1A). The DS of ‘IR64’ was 7.6 and those of the six IR64-PYLs ranged from 2.8 to 5.0. The DSs of most IR64-PYLs were significantly lower than those of the corresponding IR64-NILs, excepting IR64-*BPH32* + *BPH17*. We also evaluated the DSs of IR64-PYLs against Koshi-2020 (Fig. 3). The DS of ‘IR64’ was 8.2 and those of the six PYLs ranged from 4.2 to 5.5. The DSs of most of IR64-PYLs were significantly lower than those of the corresponding NILs. However, the DSs of IR64-*BPH3* + *BPH17* and IR64-*BPH32* + *BPH17* were significantly lower than those of one corresponding IR64-NIL but not the other NIL.

### Confirmation of the presence of *BPH17* in the PYLs

IR64-*BPH3* + *BPH17* and IR64-*BPH32* + *BPH17* had higher resistance than the other PYLs against current strongly virulent BPH populations. We confirmed the presence of *BPH17* from ‘Rathu Heenati’ in the two PYLs by PCR amplification of two InDel markers (Supplemental Figs. 2, 3). Both PYLs showed the same DNA band as ‘Rathu Heenati’.

### Agronomic traits of the IR64-PYLs

In general, the agronomic traits of the IR64-PYLs were similar to those of ‘IR64’. PL, LL, and PN did not differ significantly (Table 4). DTH, CL, and LW were also similar to those of ‘IR64’, with the exception of DTH of IR64-*BPH3* + *BPH17* and IR64-*BPH32* + *BPH17*, and CL and LW of both IR64-*BPH32* + *BPH20* and IR64-*BPH20* + *BPH3*.

## Discussion

Several BPH-resistant varieties and lines have been developed through the introgression of resistance genes for the eco-friendly management of BPH. But most older BPH-resistant varieties have only single resistance genes, which BPH overcomes rapidly, within a few years of release (Alam and Cohen 1998, Cohen *et al.* 1997). Therefore, it is crucial to develop varieties with multiple BPH resistance genes for effective control of BPH. To confirm the effect and interaction of each gene, NILs and PYLs with BPH resistance genes have been developed in the genetic backgrounds of susceptible variety (Han *et al.* 2018, Hu *et al.* 2013, Liu *et al.* 2016, Nguyen *et al.* 2019). For example, Nguyen *et al.* (2019) characterized resistance level of NILs and PYLs in the susceptible ‘T65’ background to understand effects of each gene. On the other hand, Kamal *et al.* (2023) developed NILs in the genetic background of resistance variety ‘IR64’ for the estimation of useful combination of BPH resistance genes against BPH population with strong virulence. Here, to confirm effectiveness of resistance genes those has lost its effect, we developed PYLs with four resistance genes in the elite variety ‘IR64’ genetic background, which already has *BPH1* and *BPH37*. This allowed the efficient development of PYLs with four resistance genes in ‘IR64’ background.

Several studies have shown that pyramiding of multiple resistance genes can increase resistance to BPH in rice. PYLs carrying *BPH14* and *BPH15* had higher resistance than the corresponding NILs (Hu *et al.* 2012), and a PYL carrying *BPH3* and *BPH27(t)* had significantly higher resistance (Liu *et al.* 2016). Pyramiding of *BPH6* and *BPH12*, as well as of *BPH25* and *BPH26*, also resulted in higher resistance than in corresponding NILs (Myint *et al.* 2012, Qiu *et al.* 2012). Here, IR64-PYLs had higher resistance than the corresponding NILs (Table 2, Figs. 2, 3), and BPH on these PYLs excreted less honeydew than on the corresponding NILs, suggesting that the IR64-PYLs inhibit the sucking of phloem sap from the plant (Table 3, Supplemental Fig. 1B). IR64-*BPH32* + *BPH20*, IR64-*BPH3* + *BPH17-ptb*, IR64-*BPH20* + *BPH3*, and IR64-*BPH17-ptb* + *BPH32* showed additive effects of gene pyramiding, with increased resistance to both Koshi-2013 and Koshi-2020 (Figs. 2, 3, Supplemental Fig. 1A). The resistance of IR64-*BPH3* + *BPH17* and IR64-*BPH32* + *BPH17* was close to that of ‘Rathu Heenati’ against both populations because of the strong effect of *BPH17*. However, the pyramiding effects were masked by *BPH17*, which is a cluster of three genes (*OsLecRK1–3*), which might work together to provide broad-spectrum and durable resistance to BPH (Liu *et al.* 2015). The results suggest that these combinations of genes in PYLs control strongly virulent BPH populations such as Koshi-2013 and Koshi-2020.

Overall, the virulence of Koshi-2020 was slightly higher than that of Koshi-2013 in antibiosis test and MSST. Nguyen *et al.* (2019) reported that Koshi-2013 was more virulent than Hadano-1966. Testing the long-term virulence of BPH populations to several varieties. Fujii *et al.* (2021) found that it became stronger year by year. The resistance of ‘Rathu Heenati’ and ‘Balamawee’ was generally the strongest, but it was slightly decreased against a 2019 population (Fujii *et al.* 2021). Here, the resistance of ‘Rathu Heenati’ and PYLs to Koshi-2020 was slightly lower than that to Koshi-2013. These facts suggest that the virulence of BPH increased from 2013 to 2020. We developed six PYLs in ‘IR64’ with a total of four resistance genes. These lines might be useful in monitoring strongly virulent migratory BPH stains and might also increase the durability of host-plant resistance to BPH through the development of multiline cultivars.

## Author Contribution Statement

MMK and DF designed the study. MMK, CDN, and DF developed the plant materials. SS-M reared the insects. SZ supported the research and wrote the manuscript. MMK and DF performed the experiments and wrote the manuscript.

## Supplementary Material

Supplemental Figures

Supplemental Tables

## Figures and Tables

**Table 1.** Development of pyramided lines for BPH resistance in Indica Group variety ‘IR64’ genetic background

PYL	Gene*^a^*	Donor	Generation of PYLs*^b^*
IR64-*BPH17-ptb* + *BPH32*	*BPH32* (6)	PTB33	F_3_ (BC_3_F_2_)
	*BPH17-ptb* (4)	PTB33	
IR64-*BPH32* + *BPH17*	*BPH32* (6)	PTB33	F_3_ (BC_3_F_2_)
	*BPH17* (4)	Rathu Heenati	
IR64-*BPH32* + *BPH20*	*BPH32* (6)	PTB33	F_3_ (BC_3_F_2_)
	*BPH20* (4)	IR71033-121-15	
IR64-*BPH3* + *BPH17-ptb*	*BPH3* (6)	Rathu Heenati	F_3_ (BC_3_F_2_)
	*BPH17-ptb* (4)	PTB33	
IR64-*BPH3* + *BPH17*	*BPH3* (6)	Rathu Heenati	F_3_ (BC_3_F_2_)
	*BPH17* (4)	Rathu Heenati	
IR64-*BPH20* + *BPH3*	*BPH3* (6)	Rathu Heenati	F_3_ (BC_3_F_2_)
	*BPH20* (4)	IR71033-121-15	

*^a^* Numbers in parentheses indicate chromosome number. *^b^* Generations of PYLs shown in parentheses are the generations of the NILs used as parents.

**Table 2.** Adult mortality (%) of Koshi-2013 and Koshi-2020 in antibiosis test at 5 days after infestation on IR64-PYLs

Entry	Adult mortality (%) (Mean value ± SD)	
	Koshi-2013	Koshi-2020
IR64-*BPH32*	72.0 ± 10.9 ^bc^	70.0 ± 11.5 ^ab^
IR64-*BPH17-ptb*	68.0 ± 10.9 ^cd^	65.0 ± 19.1 ^ab^
IR64-*BPH20*	80.0 ± 14.1 ^abc^	65.0 ± 10.0 ^ab^
IR64-*BPH17*	92.0 ± 10.9 ^ab^	80.0 ± 16.3 ^ab^
IR64-*BPH3*	88.0 ± 10.9 ^abc^	70.0 ± 25.8 ^ab^
IR64-*BPH3* + *BPH17*	100.0 ± 0.0 ^a^	90.0 ± 11.5 ^a^
IR64-*BPH32* + *BPH17*	100.0 ± 0.0 ^a^	95.0 ± 10.0 ^a^
IR64-*BPH32* + *BPH20*	92.0 ± 10.9 ^ab^	90.0 ± 11.5 ^a^
IR64-*BPH3* + *BPH17-ptb*	100.0 ± 0.0 ^a^	80.0 ± 16.3 ^ab^
IR64-*BPH20* + *BPH3*	96.0 ± 8.9 ^a^	80.0 ± 16.3 ^ab^
IR64-*BPH17-ptb* + *BPH32*	96.0 ± 8.9 ^a^	85.0 ± 19.1 ^a^
Rathu Heenati	100.0 ± 0.0 ^a^	95.0 ± 10.0 ^a^
IR64	48.0 ± 17.9 ^d^	45.0 ± 10.0 ^b^
Taichung 65	4.0 ± 8.9 ^e^	5.0 ± 10.0 ^c^

Values with the same letter are not significantly different at *P* < 0.05 by Tukey–Kramer test.

**Table 3.** Areas of honeydew (mm^2^) excreted by BPH feeding (Koshi-2013 and Koshi-2020) in antibiosis test at 24 h after infestation on IR64-PYLs

Entry	Honeydew area (mm^2^) (Mean value ± SD)	
	Koshi-2013	Koshi-2020
IR64-*BPH32*	23.7 ± 16.9 ^bcd^	19.6 ± 8.6 ^b^
IR64-*BPH17-ptb*	38.5 ± 19.1 ^b^	32.6 ± 9.7 ^b^
IR64-*BPH20*	20.6 ± 9.3 ^bcd^	16.1 ± 12.5 ^b^
IR64-*BPH17*	5.3 ± 2.1 ^d^	7.9 ± 2.4 ^b^
IR64-*BPH3*	18.4 ± 15.9 ^bcd^	16.7 ± 2.5 ^b^
IR64-*BPH3* + *BPH17*	2.6 ± 2.2 ^d^	6.0 ± 4.7 ^b^
IR64-*BPH32* + *BPH17*	3.6 ± 3.2 ^d^	6.6 ± 1.2 ^b^
IR64-*BPH32* + *BPH20*	3.3 ± 1.3 ^d^	9.6 ± 2.0 ^b^
IR64-*BPH3* + *BPH17-ptb*	4.2 ± 1.9 ^d^	9.6 ± 6.9 ^b^
IR64-*BPH20* + *BPH3*	4.9 ± 4.1 ^d^	8.8 ± 2.1 ^b^
IR64-*BPH17-ptb* + *BPH32*	6.2 ± 5.6 ^cd^	8.4 ± 3.5 ^b^
Rathu Heenati	3.2 ± 2.2 ^d^	7.4 ± 2.4 ^b^
IR64	26.3 ± 9.6 ^bc^	28.4 ± 13.6 ^b^
Taichung 65	79.9 ± 20.3 ^a^	83.8 ± 24.1 ^a^

Values with the same letter are not significantly different at *P* < 0.05 by Tukey–Kramer test.

**Table 4.** Agronomic traits of near-isogenic lines and pyramided lines carrying BPH resistance genes

Line	Agronomic trait (mean ± SD)					
	DTH	CL (cm)	PL (cm)	LL (cm)	LW (cm)	PN
IR64-*BPH32*	101.0 ± 1.3	89.3 ± 5.3**	24.8 ± 1.2*	24.2 ± 2.3	1.5 ± 0.1**	16.1 ± 2.7
IR64-*BPH17-ptb*	101.6 ± 0.9	92.6 ± 2.2	26.1 ± 1.4	25.7 ± 2.1	1.6 ± 0.1	15.3 ± 1.7
IR64-*BPH20*	96.4 ± 1.4***	88.5 ± 4.3**	26.8 ± 0.7	24.8 ± 2.6	1.6 ± 0.1	15.7 ± 2.1
IR64-*BPH17*	98.2 ± 1.3***	91.1 ± 3.0	26.6 ± 1.2	24.0 ± 1.7	1.5 ± 0.1	15.4 ± 1.9
IR64-*BPH3*	100.9 ± 2.0	95.4 ± 1.8	26.2 ± 1.2	27.0 ± 2.5	1.6 ± 0.1	16.8 ± 1.9
IR64-*BPH3* + *BPH17*	99.4 ± 1.3**	92.4 ± 3.8	26.5 ± 0.9	25.3 ± 2.3	1.6 ± 0.1	16.7 ± 2.1
IR64-*BPH32* + *BPH17*	99.5 ± 2.0**	92.1 ± 5.5	27.0 ± 1.2	27.1 ± 2.6	1.6 ± 0.1	15.2 ± 1.8
IR64-*BPH32* + *BPH20*	101.2 ± 2.5	89.8 ± 5.7**	25.2 ± 1.9	24.6 ± 3.4	1.4 ± 0.1***	16.7 ± 2.8
IR64-*BPH3* + *BPH17-ptb*	102.3 ± 0.5	91.6 ± 3.7	27.0 ± 1.1	27.7 ± 3.2	1.6 ± 0.1	15.7 ± 2.6
IR64-*BPH20* + *BPH3*	101.3 ± 1.5	89.9 ± 6.1*	26.9 ± 1.4	25.1 ± 3.9	1.5 ± 0.2*	16.3 ± 2.3
IR64-*BPH17-ptb* + *BPH32*	101.9 ± 0.8	92.2 ± 4.3	26.9 ± 1.4	27.3 ± 2.2	1.6 ± 0.1	15.3 ± 1.9
IR64	101.0 ± 1.5	93.7 ± 3.1	26.1 ± 1.2	25.4 ± 3.5	1.6 ± 0.1	16.6 ± 2.8

DTH, days to heading; CL, culm length; PL, panicle length; LL, flag leaf length; LW, flag leaf width; PN, panicle number; * *P* < 0.05, ** *P* < 0.01, *** *P* < 0.001 (Dunnett’s multiple comparison tests against ‘IR64’).

## References

[B1] Alam, S.N. and M.B. Cohen (1998) Durability of brown planthopper, *Nilaparvata lugens*, resistance in rice variety IR64 in greenhouse selection studies. Entomol Exp Appl 89: 71–78.

[B2] Cohen, M.B., S.N. Alam, E.B. Medina and C.C. Bernal (1997) Brown planthopper, *Nilaparvata lugens*, resistance in rice cultivar IR64: Mechanism and role in successful *N. lugens* management in Central Luzon, Philippines. Entomol Exp Appl 85: 221–229.

[B3] Dellaporta, S.L., J. Wood and J.B. Hicks (1983) A plant DNA minipreparation: Version II. Plant Mol Biol Report 1: 19–21.

[B4] Fujii, T., K. Yoshida, T. Kobayashi, K.K.M. Myint, H. Yasui, S. Sanada-Morimura and M. Matsumura (2021) Long-term virulence monitoring of differential cultivars in Japan’s immigrant populations of *Nilaparvata lugens* (Hemiptera: Delphacidae) in 2001–2019. Appl Entomol Zool 56: 407–418.

[B5] Han, Y., C. Wu, L. Yang, D. Zhang and Y. Xiao (2018) Resistance to *Nilaparvata lugens* in rice lines introgressed with the resistance genes *Bph14* and *Bph15* and related resistance types. PLoS One 13: e0198630. 29856853 10.1371/journal.pone.0198630 PMC5983517

[B6] He, L., L. Zou, Q. Huang, X. Sheng, W. Wu and J. Hu (2020) Development of InDel markers of *Bph3* and pyramiding of four brown planthopper resistance genes into an elite rice variety. Mol Breed 40: 95.

[B7] Heinrichs, E.A., F.G. Medrano and H.R. Rapusas (1985) Brown planthopper, whitebacked planthopper, green leafhopper, and zigzag leafhopper. *In*: Heinrichs, E.A., F.G. Medrano and H.R. Rapusas (eds.) Genetic evaluation for insect resistance in rice, International Rice Research Institute, Los Baños, pp. 71–170.

[B8] Horgan, F.G. (2018) Integrating gene deployment and crop management for improved rice resistance to Asian planthoppers. Crop Prot 110: 21–33.

[B9] Horgan, F.G., A.F. Ramal, J.S. Bentur, R. Kumar, K.V. Bhanu, P.S. Sarao, E.H. Iswanto, H.V. Chien, M.H. Phyu, C.C. Bernal et al. (2015) Virulence of brown planthopper (*Nilaparvata lugens*) populations from South and South East Asia against resistant rice varieties. Crop Prot 78: 222–231.

[B10] Hu, J., X. Li, C. Wu, C. Yang, H. Hua, G. Gao, J. Xiao and Y. He (2012) Pyramiding and evaluation of the brown planthopper resistance genes *Bph14* and *Bph15* in hybrid rice. Mol Breed 29: 61–69.

[B11] Hu, J., M. Cheng, G. Gao, Q. Zhang, J. Xiao and Y. He (2013) Pyramiding and evaluation of three dominant brown planthopper resistance genes in the elite *indica* rice 9311 and its hybrids. Pest Manag Sci 69: 802–808. 23175467 10.1002/ps.3437

[B12] Hu, W., H. Xiao, K. Hu, Y. Jiang and Y. Zhang (2016) Application of marker-assisted backcross to introgress *Bph3*, *Bph14* and *Bph15* into an elite *indica* rice variety for improving its resistance to brown planthopper. Plant Breed 135: 291–300.

[B13] IRRI (International Rice Research Institute) (2014) Standard evaluation system for rice (SES) 5th edn. International Rice Research Institute, Los Baños, p. 57.

[B14] Jena, K.K., S.L. Hechanova, H. Verdeprado, G.D. Prahalada and S.R. Kim (2017) Development of 25 near-isogenic lines (NILs) with ten BPH resistance genes in rice (*Oryza sativa* L.): Production, resistance spectrum, and molecular analysis. Theor Appl Genet 130: 2345–2360. 28795219 10.1007/s00122-017-2963-8

[B15] Kamal, M.M., C.D. Nguyen, S. Sanada-Morimura, S.H. Zheng and D. Fujita (2023) Near-isogenic lines for resistance to brown planthopper with the genetic background of Indica Group elite rice (*Oryza sativa* L.) variety ‘IR64’. Breed Sci 73: 278–289. 37840984 10.1270/jsbbs.22093 PMC10570883

[B16] Khush, G.S. and P.S. Virk (2005) IR varieties and their impact. *In*: Hardy, B. (ed.) Selection criteria, International Rice Research Institute, Los Baños, pp. 6–15.

[B17] Li, Z., Y. Xue, H. Zhou, Y. Li, B. Usman, X. Jiao, X. Wang, F. Liu, B. Qin, R. Li et al. (2019) High-resolution mapping and breeding application of a novel brown planthopper resistance gene derived from wild rice (*Oryza. rufipogon* Griff). Rice (N Y) 12: 41. 31165331 10.1186/s12284-019-0289-7 PMC6548798

[B18] Liu, Y., H. Wu, H. Chen, Y. Liu, J. He, H. Kang, Z. Sun, G. Pan, Q. Wang, J. Hu et al. (2015) A gene cluster encoding lectin receptor kinases confers broad-spectrum and durable insect resistance in rice. Nat Biotechol 33: 301–305. 10.1038/nbt.3069 25485617

[B19] Liu, Y., L. Chen, Y. Liu, H. Dai, J. He, H. Kang, G. Pan, J. Huang, Z. Qiu, Q. Wang et al. (2016) Marker assisted pyramiding of two brown planthopper resistance genes, *Bph3* and *Bph27*(*t*), into elite rice cultivars. Rice (N Y) 9: 27. 27246014 10.1186/s12284-016-0096-3 PMC4887400

[B20] Myint, K.K.M., H. Yasui, M. Takagi and M. Matsumura (2009) Virulence of long-term laboratory populations of the brown planthopper, *Nilaparvata lugens* (Stål), and whitebacked planthopper, *Sogatella furcifera* (Horváth) (Homoptera: Delphacidae), on rice differential varieties. Appl Entomol Zool 44: 149–153.

[B21] Myint, K.K.M., D. Fujita, M. Matsumura, T. Sonoda, A. Yoshimura and H. Yasui (2012) Mapping and pyramiding of two major genes for resistance to the brown planthopper (*Nilaparvata lugens* [Stål]) in the rice cultivar ADR52. Theor Appl Genet 124: 495–504. 22048639 10.1007/s00122-011-1723-4 PMC3265730

[B22] Nguyen, C.D., H. Verdeprado, D. Zita, S. Sanada-Morimura, M. Matsumura, P.S. Virk, D.S. Brar, F.G. Horgan, H. Yasui and D. Fujita (2019) The development and characterization of near-isogenic and pyramided lines carrying resistance genes to brown planthopper with the genetic background of *japonica* rice (*Oryza sativa* L.). Plants (Basel) 8: 498. 31726710 10.3390/plants8110498 PMC6918374

[B23] Qiu, Y., J. Guo, S. Jing, L. Zhu and G. He (2012) Development and characterization of *japonica* rice lines carrying the brown planthopper-resistance genes *BPH12* and *BPH6*. Theor Appl Genet 124: 485–494. 22038433 10.1007/s00122-011-1722-5

[B24] Wei, J., D. Jia, Q. Mao, X. Zhang, Q. Chen, W. Wu, H. Chen and T. Wei (2018) Complex interactions between insect-borne rice viruses and their vectors. Curr Opin Virol 33: 18–23. 30031984 10.1016/j.coviro.2018.07.005

[B25] Yang, M., L. Cheng, L. Yan, W. Shu, X. Wang and Y. Qiu (2019) Mapping and characterization of a quantitative trait locus resistance to the brown planthopper in the rice variety IR64. Hereditas 156: 22. 31297040 10.1186/s41065-019-0098-4 PMC6595561

