# Supplementary material for: Development of pyramided lines carrying brown planthopper resistance genes in the genetic background of Indica Group rice (Oryza sativa L.) variety ‘IR64’
Source: Breed Sci. 2023 Oct 31;73(5):450–6. doi: 10.1270/jsbbs.23028 (PMC11082456; doi:10.1270/jsbbs.23028)
Supplement: Supplementary file 1 — Supplemental Figures [file 73_450_s1.pdf]

doi: 10.1270/jsbbs.23028

A

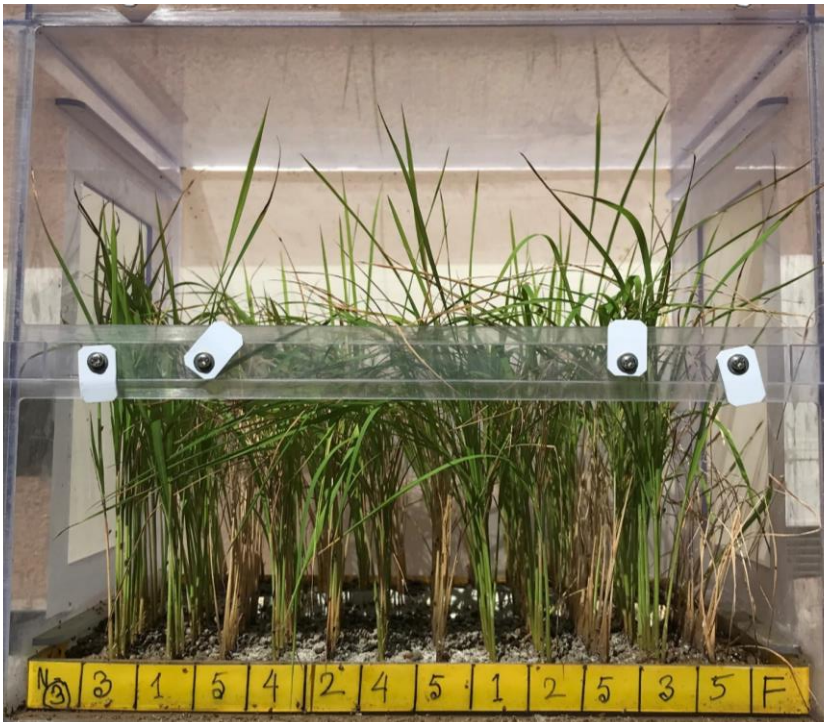

1. IR64-*BPH17*-NIL
2. IR64-*BPH3*-NIL
3. IR64-*BPH3*+*BPH17*-PYL
4. IR64
5. Taichung 65

B

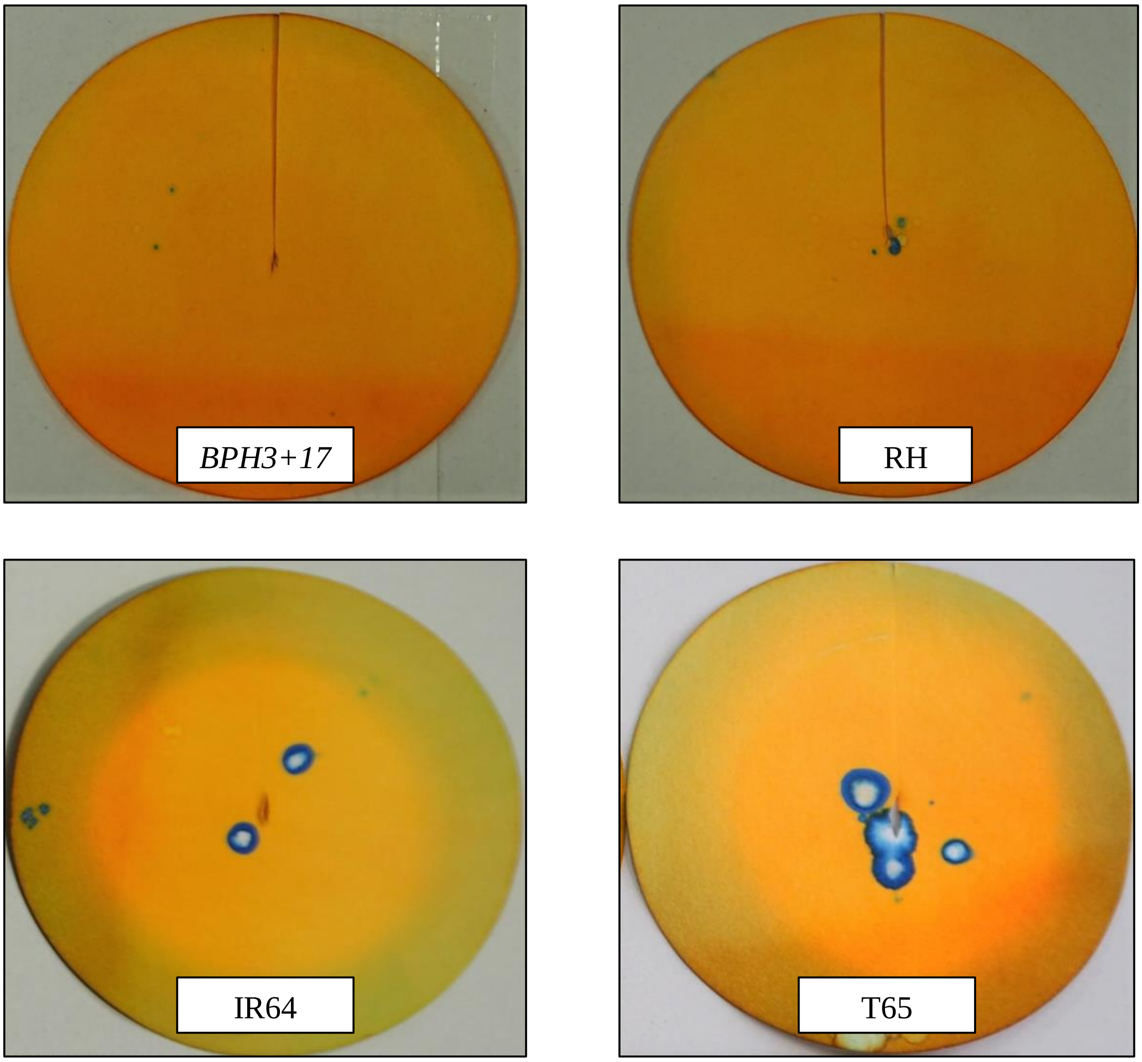

Supplemental Fig. 1 (A) Modified seed box screening test and (B) honeydew test of Koshi-2013 on PYLs carrying BPH resistance genes.

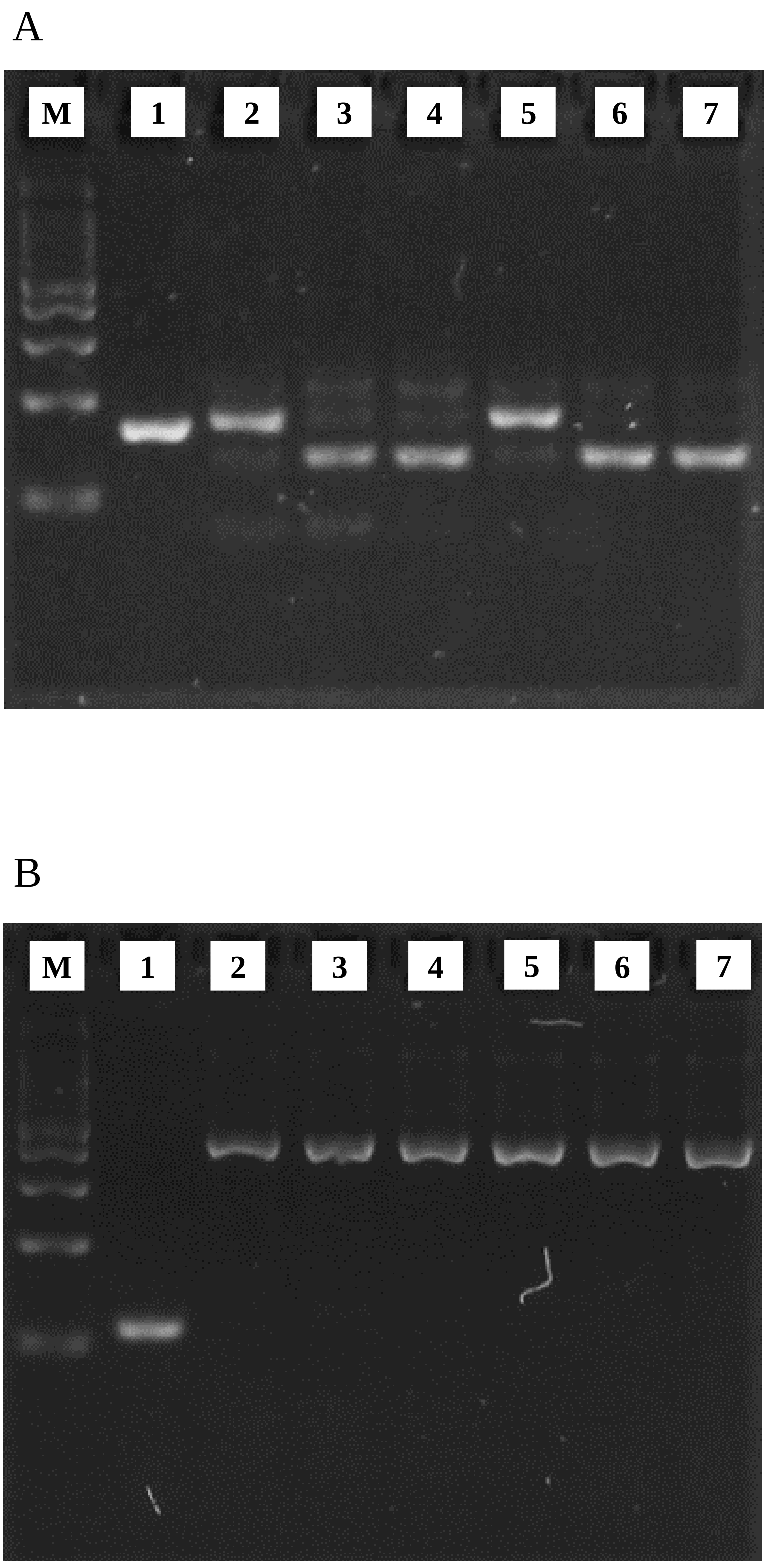

Supplemental Fig. 2 Confirmation of *BPH17* resistance genes from IR64-NIL using (A) I531 and (B) I729 InDel markers [lanes: 1, IR64; 2, IR64-*BPH17*-NIL (RH); 3, IR64-*BPH17-ptb*-NIL (PTB33); 4, IR64-*BPH20*-NIL (IR71033-122-15); 5, RH, 6, PTB33; 7, IR71033-122-15; M, DNA ladder].

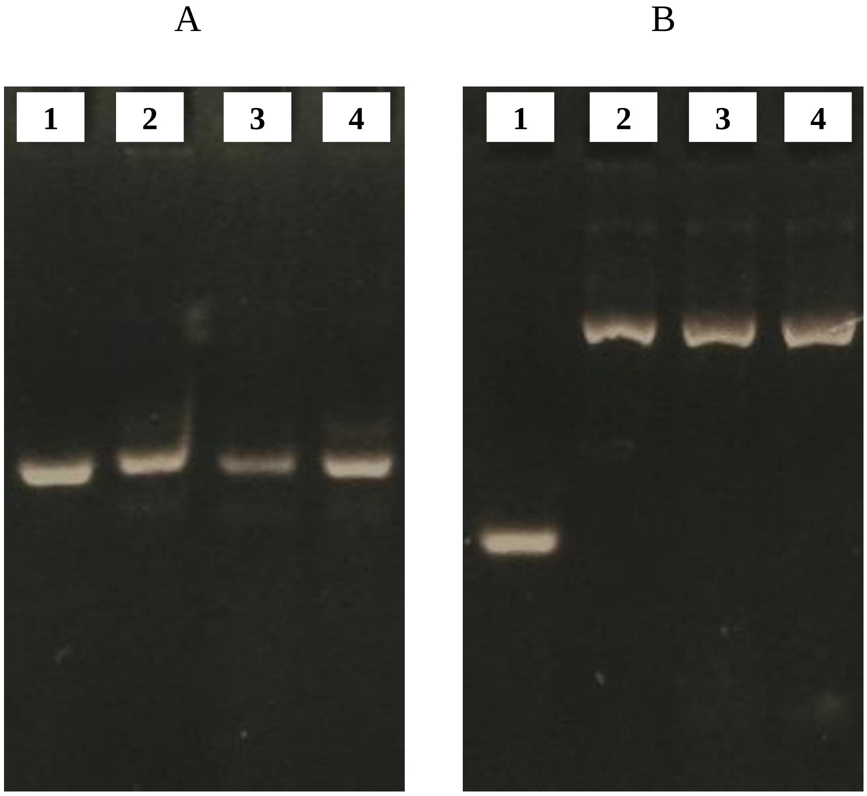

Supplemental Fig. 3 Confirmation of *BPH17* resistance genes from IR64-PYLs using (A) I531 and (B) I729 InDel markers [lanes: 1, IR64; 2, IR64-*BPH17*+*BPH32*- PYL; 3, IR64-*BPH17*+*BPH3*-PYL; 4, RH].
