# Supplementary material for: Development of pyramided lines carrying brown planthopper resistance genes in the genetic background of Indica Group rice (Oryza sativa L.) variety ‘IR64’
Source: Breed Sci. 2023 Oct 31;73(5):450–6. doi: 10.1270/jsbbs.23028 (PMC11082456; doi:10.1270/jsbbs.23028)
Supplement: Supplementary file 2 — Supplemental Tables [file 73_450_s2.pdf]

doi: 10.1270/jsbbs.23028

Supplemental Table 1. The SSR markers used for marker-assisted selection (MAS) of plants carrying BPH resistance genes from the resistance donors

Marker Name	Chromosome	Resistance gene (s)	Forward primer sequence (5′→3′)	Reverse Primer Sequence (5′→3′)	Physical Position (Mbp) *	References
RM508	6	*BPH3, BPH32*	AGAAGCCGGTTCATAGTTCATGC	ACCCGTGAACCACAAAGAACG	0.44	Temnykh *et al.* (2001)
RM19341	6	*BPH32*	GCTACAAATAGCCACCCACACC	CAACACAAGCAGAGAAGTGAAGC	1.76	IRGSP (2005)
RM588	6	*BPH3*	TCTTGCTGTGCTGTTAGTGTACG	GCAGGACATAAATACTAGGCATGG	1.61	Temnykh *et al.* (2001)
RM8213	4	*BPH17, BPH17-ptb, BPH20*	TGTTGGGTGGGTAAAGTAGATGC	CCCAGTGATACAAAGATGAGTTGG	4.42	McCouch *et al*. (2002)
MS10	4	*BPH17, BPH17-ptb, BPH20*	CAATACGAGAAGCCCCTCAC	CTGAAGGAACACGCGGTAGT	8.08	Rahman *et al*. (2009), Yang *et al*. (2004)
RM16535	4	*BPH17, BPH17-ptb, BPH20*	ACGCGGTAGTCCTCTTCAATGTCG	GGCGCCAACCCTTCCTACTACC	8.03	IRGSP (2005)

* The physical position of marker was obtained from Nipponbare genome sequence in IRGSP ver1.0.

Supplemental Literature Cited

IRGSP (International Rice Genome Sequencing Project) (2005) The map-based sequence of the rice genome. Nature 436: 793–800.

McCouch, S.R., L. Teytelman, Y. Xu, K.B. Lobos, K. Clare, M. Walton, B. Fu, R. Maghirang, Z. Li, Y. Xing *et al*. (2002) Development and mapping of 2240 new SSR markers for rice (*Oryza sativa* L.). DNA Res 9: 199–207.

Rahman, M.L., W. Jiang, S.H. Chu, Y. Qiao, T.H. Ham, M.O. Woo, J. Lee, M.S. Khanam, J.H. Chin, J.U. Jeung *et al*. (2009) High-resolution mapping of two rice brown planthopper resistance genes, *Bph20* (t) and *Bph21* (t), originating from *Oryza minuta*. Theor Appl Genet 119: 1237–1246.

Temnykh, S., G. DeClerck, A. Lukashova, L. Lipovich, S. Cartinhour and S. McCouch (2001) Computational and experimental analysis of microsatellites in rice (*Oryza sativa* L.): Frequency, length variation, transposon associations, and genetic marker potential. Genome Res 11: 1441–1452.

Yang, H., A. You, Z. Yang, F. Zhang, R. He, L. Zhu and G. He (2004) High-resolution genetic mapping at the *Bph15* locus for brown planthopper resistance in rice (*Oryza sativa* L.). Theor Appl Genet 110: 182–191.

Supplemental Table 2. The InDel markers used for the confirmation of *BPH17* resistance genes on chromosome 4 in the developed NILs and PYLs

Marker Name	ID	Forward primer sequence (5′→3′)	Reverse Primer Sequence (5′→3′)	*Var (bp)
I939[a]	vg0406931939	CGGTGGTGGATGTTCTTGAT	TTCAGCTCGATCAGATTCCA	5
I531[a]	vg0406936531	ATTTAGGCTCGTCACGGATG	GGGTTGTTGGGAAAAGGAAT	7
I729[a]	vg0406936729	CATCGACATTCACACACCCA	TGCATGTCGCTGAATGGATG	12
I106[a]	vg0406944106	ATGGGAATACCCTGGATCGGT	GATGGAGATAAGTGGCAGG	30
I350[a]	vg0406970350	CTCCATTTTAGCCATGCAC	CCGTATTCTTGTGGAAATTACC	4
I560[a]	vg0406970560	TTGGTCGATTACCATTTCCA	TAGCCATTTAACGGGGATTG	13
I818[a]	vg0406971818	GCACACCAACTGGCAACTAG	GTTACATTTTTCTTACGGC	6
I923[a]	vg0406977923	ATCACAGAGCCTCCGAGAAG	GCTCCCTCTATCAAACGGGA	3

* Var. variation (insertion or deletion) between the alleles

[a]He *et al*. (2020)
